# Comparative analysis of microbial communities and physicochemical attributes of strong-aroma Daqu from Southwestern China

**DOI:** 10.1186/s40643-025-00997-z

**Published:** 2026-01-27

**Authors:** Mingfu Shi, Aixia Guo, Yang Li, Dexiang Sun, Tao Wang, Liguo Yin

**Affiliations:** 1 Solid-State Fermentation Resource Utilization Key Laboratory of Sichuan Province, Yibin University, Yibin, 644000 Sichuan China; 2 Department of Agriculture Forestry and Food Engineering, Yibin University, Yibin, 644000 Sichuan China; 3 Institute of Agricultural Resources and Environment, Sichuan Academy of Agricultural Sciences, Chengdu, 610006 Sichuan China

**Keywords:** Daqu, Microbial communities, Physicochemical properties, Baijiu fermentation, Regional variations

## Abstract

Daqu is a critical saccharifying and fermenting agent in the traditional solid-state production of strong-aroma Baijiu, with its physicochemical properties and microbial communities playing a vital role in both starter quality and final aroma. This study analyzed twelve Daqu samples from four key production regions in Sichuan Province—Luzhou (L), Suining (S), Yibin (Y), and Zigong (Z)—assessing physicochemical parameters such as moisture content, acidity, saccharification, liquefaction, and esterification activities. Microbial communities were characterized using Illumina high-throughput sequencing. Regional variations in physicochemical properties were observed, with Y showing the highest acidity, saccharification, and esterification activities. While no significant regional differences in alpha-diversity were found, β-diversity analysis (PCoA) revealed distinct microbial structures. Bacteria were predominantly represented by *Bacillus*, *Lactobacillus*, and *Weissella*, while *Aspergillus*, *Saccharomycopsis*, and *Rhizopus* dominated fungi. LEfSe analysis (LDA ≥ 4.0) identified region-specific microbial shifts. PICRUSt2 and FUNGuild predictions indicated significant potential for amino acid and carbohydrate metabolism, with saprotrophic fungi being dominant. Correlation analysis highlighted *Thermoactinomyces*, *Weissella*,* Bacillus*, and *Rhizopus* as key microbes influencing saccharification, starch degradation, and esterification. These findings provide insights into microbial and biochemical factors driving regional differences in Daqu, offering a foundation for standardized production methods and quality control.

**Graphical abstract:**

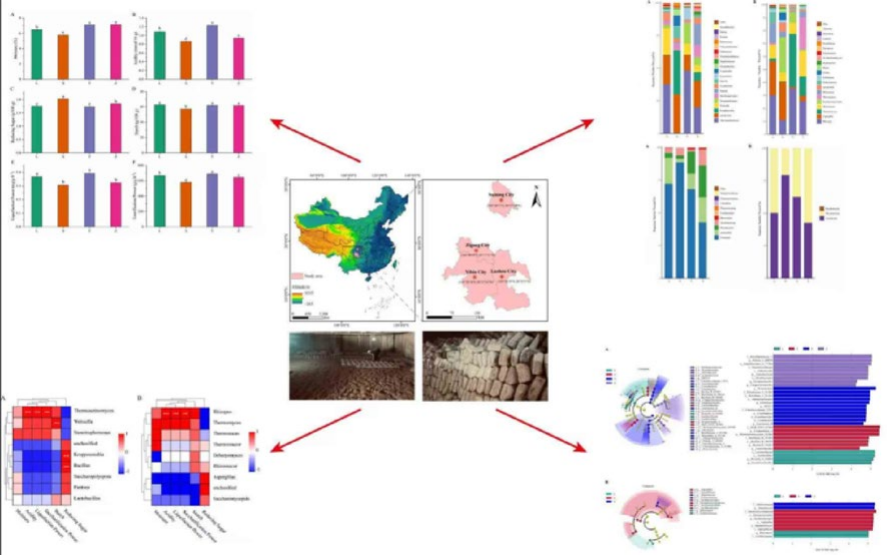

**Supplementary Information:**

The online version contains supplementary material available at 10.1186/s40643-025-00997-z.

## Introduction

### Importance of Baijiu and the strong-aroma type

**Fig. 1:**
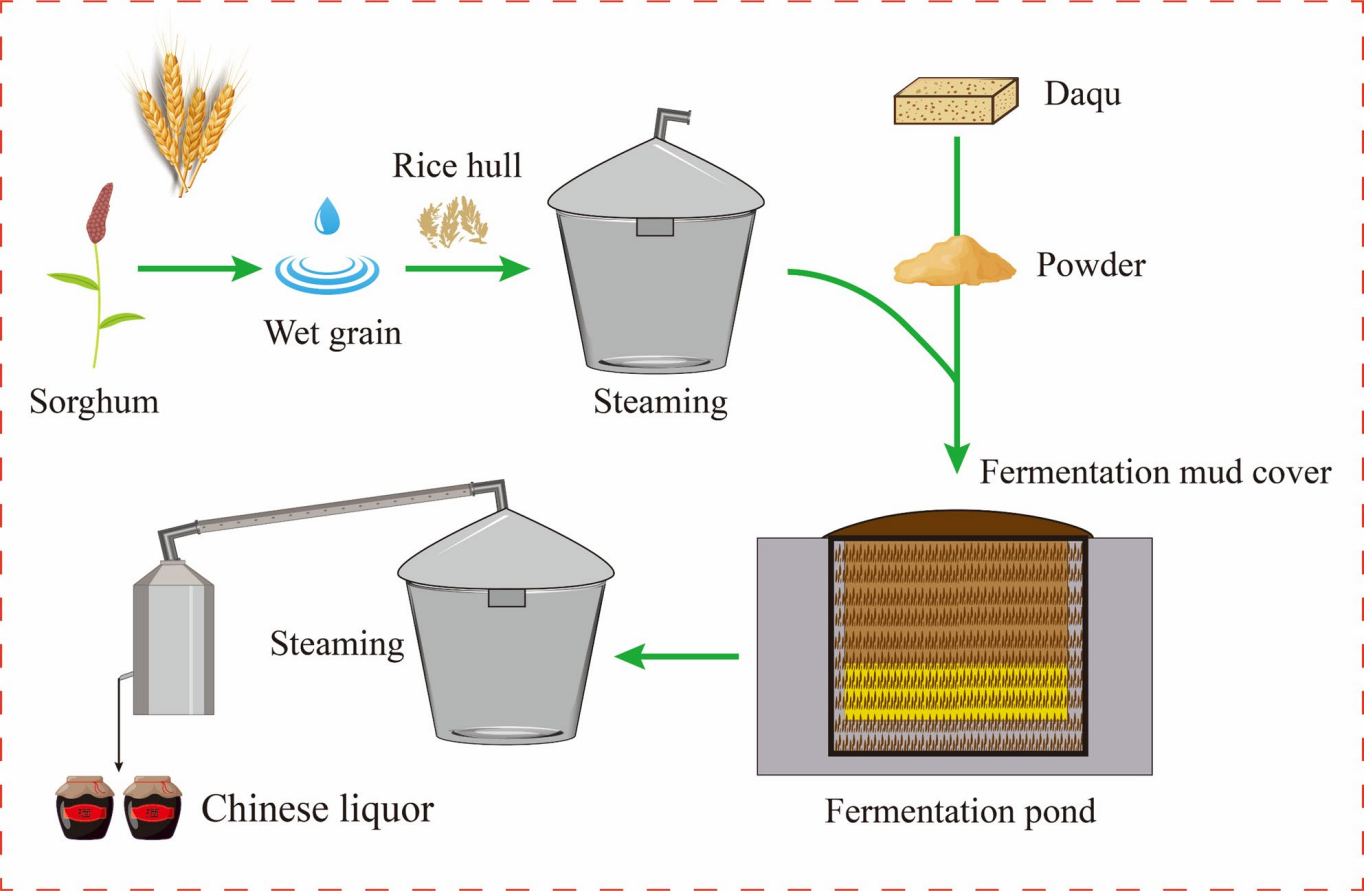
Traditional manufacturing process of strong-aroma Baijiu.

Baijiu is a traditional Chinese distilled spirit produced through solid-state fermentation of grains, and is recognized as one of the six major distilled liquors worldwide (He et al. 2019; Xu et al. 2023). Among the twelve aroma types, strong-aroma Baijiu (SAB) is the dominant category in China, accounting for nearly 50% of the total market due to its mellow, full-bodied flavor and rich ester profile (Hong et al. 2023; Jin et al. 2017). It is commonly produced in Sichuan, Jianghuai, and northern regions, with Sichuan serving as a historical and industrial hub (Wang 2022). The process typically uses sorghum as the main substrate and relies on Daqu-started, pit-based solid-state fermentation followed by distillation, aging, and blending (Chai et al. 2021; Xu et al. 2017) (He et al. 2024) (Fig. 1).

### Role of Daqu in fermentation and key challenges

Daqu is the core starter for strong-aroma Baijiu, functioning simultaneously as the enzymatic saccharification system and the living microbial inoculum that drive starch hydrolysis, alcohol formation, and early flavor generation (Deng et al. 2023; Li et al. 2017). Produced mainly from wheat (sometimes with barley, peas, or sorghum) by natural inoculation under controlled temperature and humidity, Daqu harbors diverse bacteria and fungi whose metabolic activities underpin saccharifying, liquefying, esterifying, and fermentative activities (Li et al. 2016; Wu et al. 2021). However, traditional, partially uncontrolled manufacture leads to batch-to-batch variation in microbial composition and abundance, which can propagate to fluctuations in functional indices and product quality (Ma et al. 2022a, b; Yi et al. 2024).

### Linking physicochemical properties, microbial diversity, and product quality

The physicochemical profile of Daqu—moisture, acidity, and the above functional indices—is tightly associated with its community structure and diversity (Cheng et al. 2024; Deng et al. 2025). Differences in raw materials, production temperature (high-, medium-, or low-temperature Daqu for sauce-, strong-, and light-aroma types, respectively), and local environments shape distinct community assemblies and metabolic potentials, thereby modulating aroma compound formation and fermentation robustness (Chen et al. 2025; Cui et al. 2025; Jin et al. 2017; Mu et al. 2023; Xu et al. 2017). Studies have reported correlations between dominant taxa and key indices such as starch content, moisture, and esterifying activity, and have linked community shifts to the dynamic evolution of aroma compounds during fermentation (Gong et al. 2022; Xu et al. 2017; Zhang et al. 2024a, b, c). In strong-aroma production areas—especially the Sichuan Basin with its humid climate and long fermentation tradition—Daqu and pit-mud microbiota can interact, with anaerobes from pit mud contributing to flavor formation (Hong et al. 2021; Wang et al. 2017; Xu et al. 2023). Given this variability, understanding the microbial communities and their functions is essential, and recent advances in high-throughput sequencing (HTS) offer powerful tools for such analyses.

### Research gap and objectives

Although individual reports suggest that environment, raw materials, and techniques affect Daqu communities, the microbial and functional basis of regional differences in strong-aroma Daqu produced under standardized conditions remains insufficiently resolved (Han et al. 2024; He et al. 2024; Hu et al. 2021a, b; Jin et al. 2019). Luzhou, Suining, Yibin, and Zigong are key Sichuan production centers, yet comparative studies that integrate unified sampling, physicochemical characterization, HTS-based community profiling, and function-oriented correlation analysis are scarce. Here, we collected Daqu from these four regions under standardized protocols, quantified moisture, acidity, and saccharifying, liquefying, esterifying, and fermentative activities as functional indices, profiled bacterial and fungal diversity via Illumina HTS, performed functional prediction, and constructed association networks between communities and physicochemical indices. Our objective is to elucidate regional signatures of Daqu microbial structure and function and clarify how these features co-regulate fermentation performance, thereby informing standardization, microbial resource utilization, and process optimization in strong-aroma Baijiu production.

## Materials and methods

### Sample collection

**Fig. 2:**
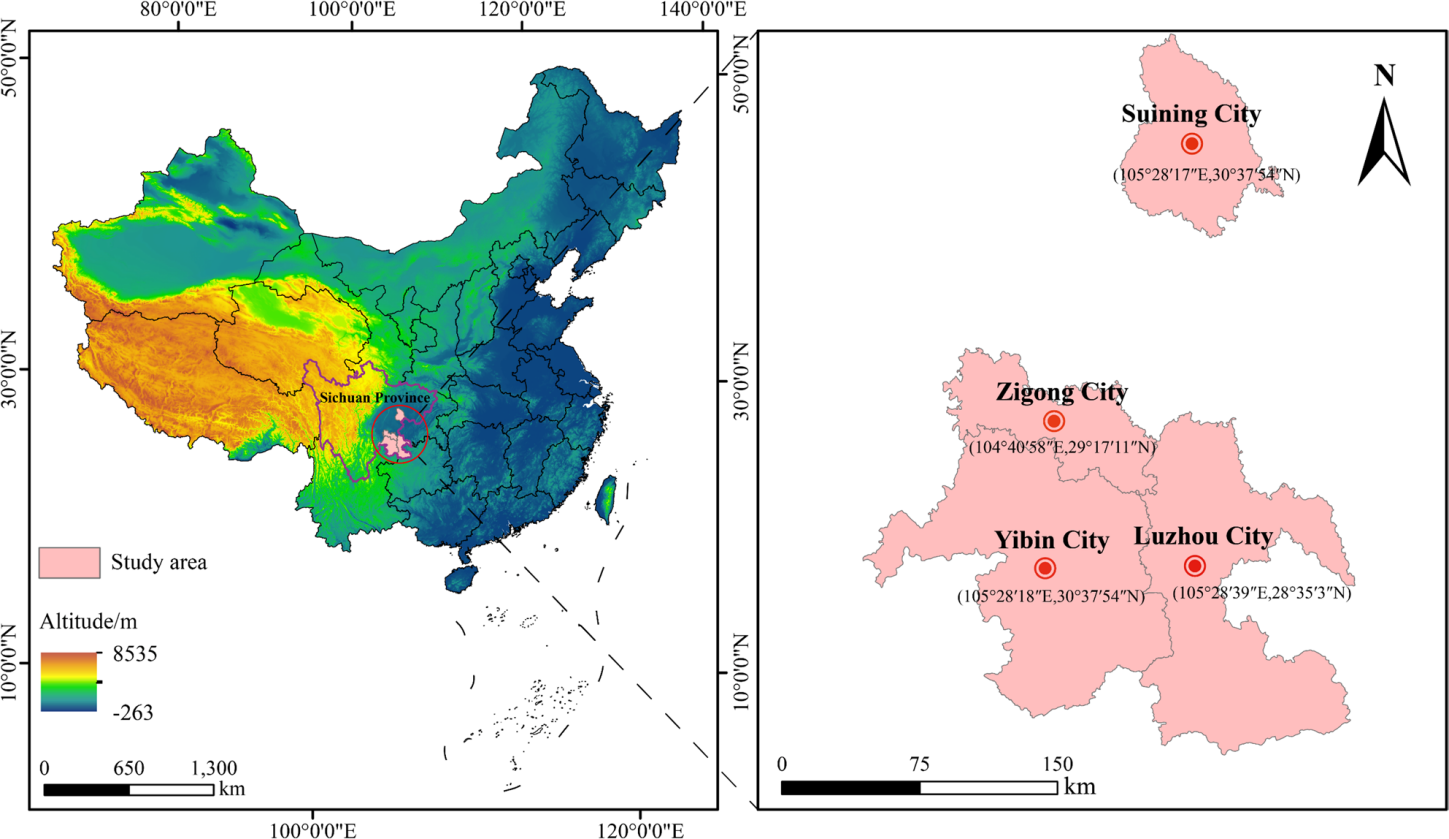
Geographical distribution of Daqu samples

Daqu was collected in April 2024 from four strong-aroma Baijiu regions in Sichuan, China: Luzhou (L), Suining (S), Yibin (Y), and Zigong (Z). For each region, three independent Daqu bricks were sampled; from each brick, subsamples were taken from the center and edge, pooled, and homogenized to form one composite sample (*n* = 3 composites per region; 12 composites total). Approximately 300 g from each composite was reserved for physicochemical assays, and the remainder was stored at − 80 °C for microbial analyses. Regional Daqu characteristics are summarized in Table S1, and sampling locations are shown in Fig. 2.

### Experimental procedures

#### Sample Preparation

Daqu samples were ground under aseptic conditions to pass through a 50-mesh sieve. A total of 300 g from each composite sample was used for physicochemical analysis, while the remaining aliquots were stored at −80 °C for microbial analysis.

#### Physicochemical analysis

Physicochemical properties were determined as follows: moisture, acidity, reducing sugar, starch, liquefaction power, and esterification power were measured according to Deng et al. 2020; Zhang et al. 2024a, b, c. Moisture was determined in triplicate using a rapid analyzer. Reducing sugar content was determined using the Fehling’s titration method as per GB/T 5009.7–2016. Starch content was measured *via* the acid hydrolysis method following GB/T 5009.9–2016. Liquefaction power, and esterification power were analyzed based on QB/T 4257 − 2011.

### Microbial community analysis

#### DNA extraction and amplicon sequencing

Genomic DNA was extracted using the PowerSoil DNA Isolation Kit (Qiagen, USA) and verified *via* 1% agarose gel electrophoresis. Negative controls were included to monitor contamination: one extraction blank per batch and no-template PCR controls for each primer set; these controls were carried through library preparation and sequencing and later used to screen potential contaminants during bioinformatic filtering. The bacterial 16 S rRNA gene V3–V4 region was amplified using primers 515 F (GTGCCAGCMGCCGCGGTAA) and 806R (GGACTACHVGGGTWTCTAAT), and fungal ITS1 regions were amplified with primers ITS5-1737 F (GCATCGATGAAGAACGCAGC) and ITS2-2043R (TCCTCCGCTTATTGATATGC). Sequencing was performed on an Illumina NovaSeq 6000 platform using paired-end 250 bp reads.

### Bioinformatics and statistical analysis

Raw sequencing data were processed and quality-filtered using fastp (v0.14.1). Sequences were clustered into operational taxonomic units (OTUs) at a 97% similarity threshold, and representative OTUs were annotated using the SILVA (16 S) and UNITE (ITS1) databases (Hu et al. 2021a, b). Microbial community analysis was performed using the Wekemo Bioincloud platform (https://www.bioincloud.tech) and R v4.3.0. Physicochemical data were analyzed statistically using IBM SPSS Statistics 26.0. One-way analysis of variance (ANOVA) was performed on the physicochemical indices of Daqu using SPSS 22.0, followed by post-hoc testing with Tukey’s Honestly Significant Difference (HSD) method. Box plots were generated using Origin 2021 to visualize data distributions. All samples were analyzed in triplicate, and results are presented as mean ± standard deviation (SD). Alpha (α) diversity (observed OTUs, Shannon) and beta (β) diversity were computed. For β-diversity, abundance-based Bray-Curtis distances were used: PCoA was performed in R (v3.3.1), with visualizations via Bioincloud to show inter-sample differences, and PERMANOVA tested group-level structural significance. The linear discriminant analysis effect size (LEfSe) (LDA ≥ 4.0) method was used to identify taxa with significant abundance differences between groups, complementary univariate tests with BH-FDR were additionally applied. Bacterial functional pathways were predicted using PICRUSt2, while fungal ecological functions were predicted using FUNGuild (Gao et al. 2024).

## Results

### Physicochemical properties of Daqu samples

**Fig. 3:**
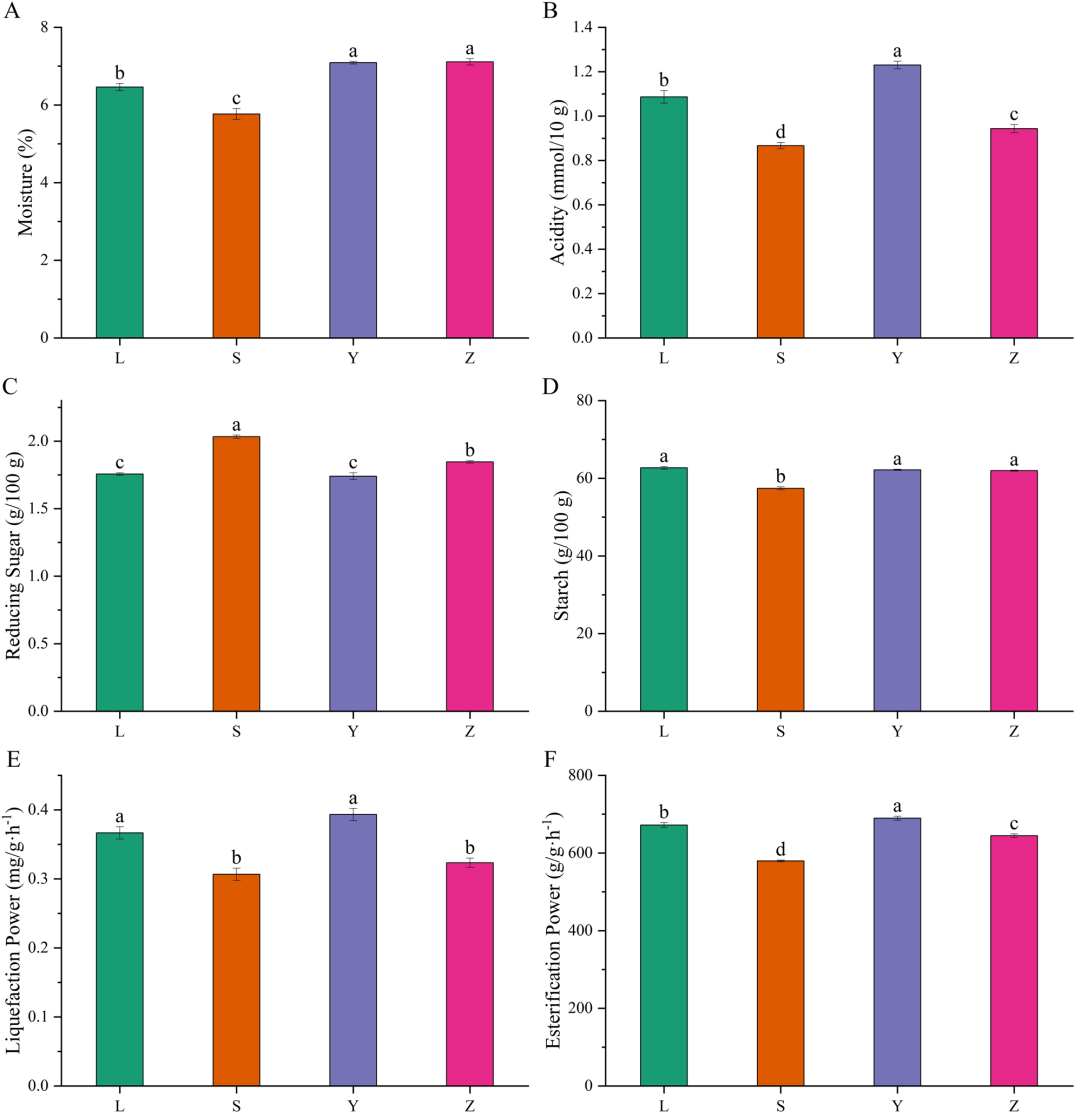
Physicochemical properties of Daqu samples from four regions. **A** Moisture Content; **B**) Acidity; **C** Reducing Sugar Content; **D** Starch Content; **E** Liquefaction Power; **F** Esterification Power. L represents Luzhou, S represents Suining, Y represents Yibin, and Z represents Zigong. Data were analyzed using one-way ANOVA followed by Tukey's HSD post-hoc test (*P* < 0.05). Different lowercase letters indicate significant differences among treatments.

The four Daqu samples (L, S, Y, and Z) exhibited significant differences in moisture content, acidity, reducing sugar, starch content, liquefaction power, and esterification power (Fig. 3). Notably, S had the lowest moisture content (5.82%), significantly lower than L (6.46%), Y (7.09%), and Z (7.11%) (Fig. 3A). For acidity, Y showed the highest value (1.23 mmol/10 g), which was significantly higher than L (0.98 mmol/10 g), S, and Z (Fig. 3B). In reducing sugar, S stood out with the highest concentration (2.03 g/100 g), significantly exceeding the other three samples (Fig. 3C). Starch content in L and Y was significantly higher than S (57.41 g/100 g), while Z was comparable to L and Y (Fig. 3D). Y exhibited the highest liquefaction power (0.39 mg/(g·h)), significantly higher than S (0.31 mg/(g·h)) and Z, with L showing no difference from Y (Fig. 3E). For esterification power, L, Y, and Z were significantly higher than S, with L having the highest value (689.54 g/(g·h); Fig. 3F). These results indicate that Daqu from different regions exhibits considerable variability in physicochemical characteristics, likely due to differences in raw materials, fermentation temperatures, and production practices.

### Sequencing quality and core Venn analysis

Rarefaction curves were used to evaluate sequencing depth and sampling adequacy. As shown in Figure S1, the curves gradually approached a plateau with increasing numbers of sequencing reads, indicating sufficient sequencing coverage. The results demonstrated that the sequencing depth captured the vast majority of bacterial (Figure S1A) and fungal (Figure S1B) species present in the samples. Only a few rare taxa remained undetected, exerting negligible influence on the overall dataset. These findings confirm that the sequencing depth and quality in this study were reasonable, reliable, and representative of the microbial diversity in the Daqu samples.

**Fig. 4:**
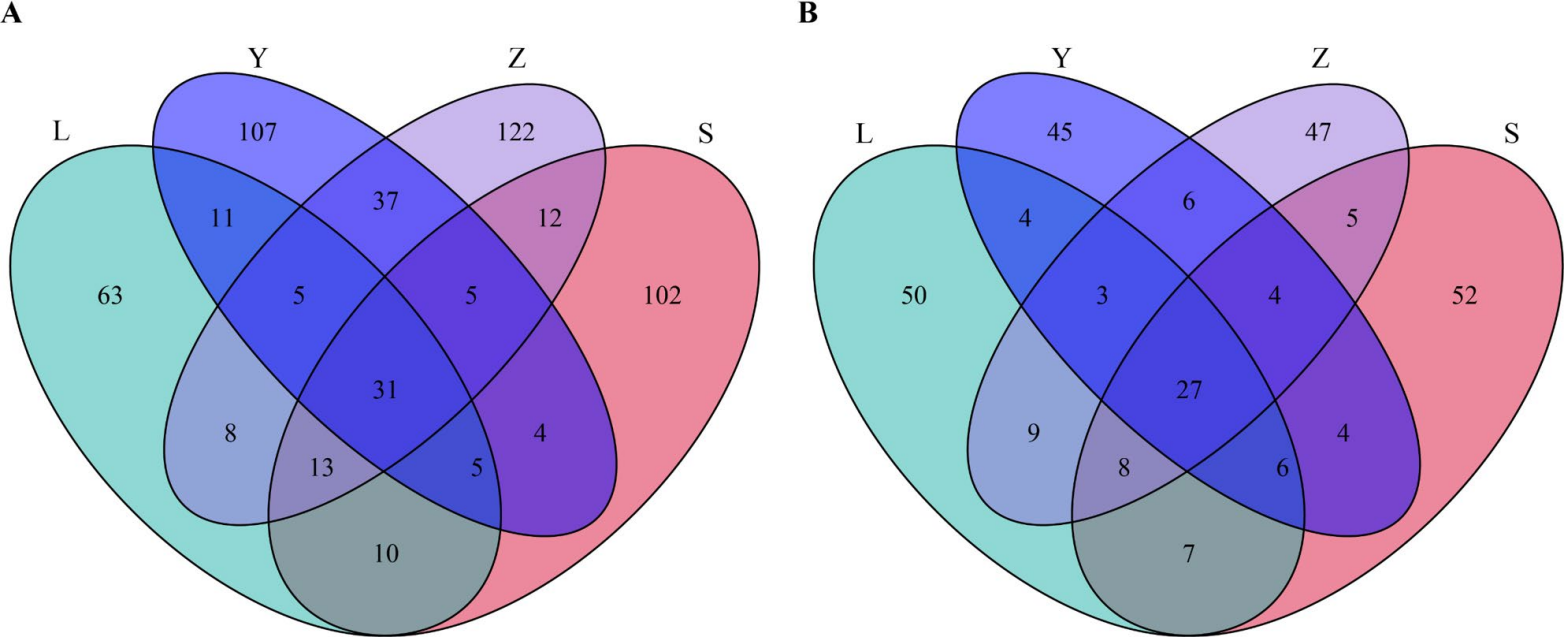
Venn diagram analysis of OTUs in Daqu samples. **A** Bacterial communities; **B** Fungal communities. L represents Luzhou, S represents Suining, Y represents Yibin, and Z represents Zigong.

To further compare shared and unique microbial taxa among regions, Venn diagrams were constructed at the genus level (Fig. 4). A total of 31 bacterial and 27 fungal genera were shared across all four regions, representing a core microbiota in Sichuan strong-aroma Daqu. In addition to the shared genera, each region contained region-specific microbial taxa. For bacteria, 63, 102, 107, and 122 unique OTUs were identified in regions L, S, Y, and Z, respectively. Similarly, 22, 40, 35, and 32 region-specific fungal OTUs were detected in regions L, S, Y, and Z, respectively.

### Alpha diversity of microbial communities in Daqu

As shown in Table 1, there were no statistically significant differences in the Chao1, Shannon, or Simpson indices among the four Daqu samples (*p* > 0.05), indicating that overall microbial richness and diversity were comparable across regions. Although no significant differences were detected, slight variations in index values were observed. For the bacterial community, sample S showed a relatively higher Chao1 index, while sample Z presented higher Shannon and Simpson indices, reflecting minor fluctuations in richness and evenness among regions. Sample Y exhibited comparatively lower alpha diversity indices. A similar overall pattern was found in the fungal community, where sample S showed relatively higher richness (Chao1), and sample Z displayed slightly higher diversity (Shannon and Simpson), whereas sample Y had lower index values.

**Table 1:** Analysis of microbial alpha diversity in Daqu

Treatment	Bacteria			Fungi		
	Chao1 index	Shannon index	Simpson index	Chao1 index	Shannon index	Simpson index
L	91.67	3.86	0.87	91.00	3.85	0.87
S	108.00	3.45	0.80	104.33	3.44	0.80
Y	64.00	2.49	0.65	64.00	2.48	0.65
Z	101.67	3.94	0.88	100.33	3.95	0.88

L represents Luzhou, S represents Suining, Y represents Yibin, and Z represents Zigong

### Beta diversity of microbial communities in Daqu

**Fig. 5:**
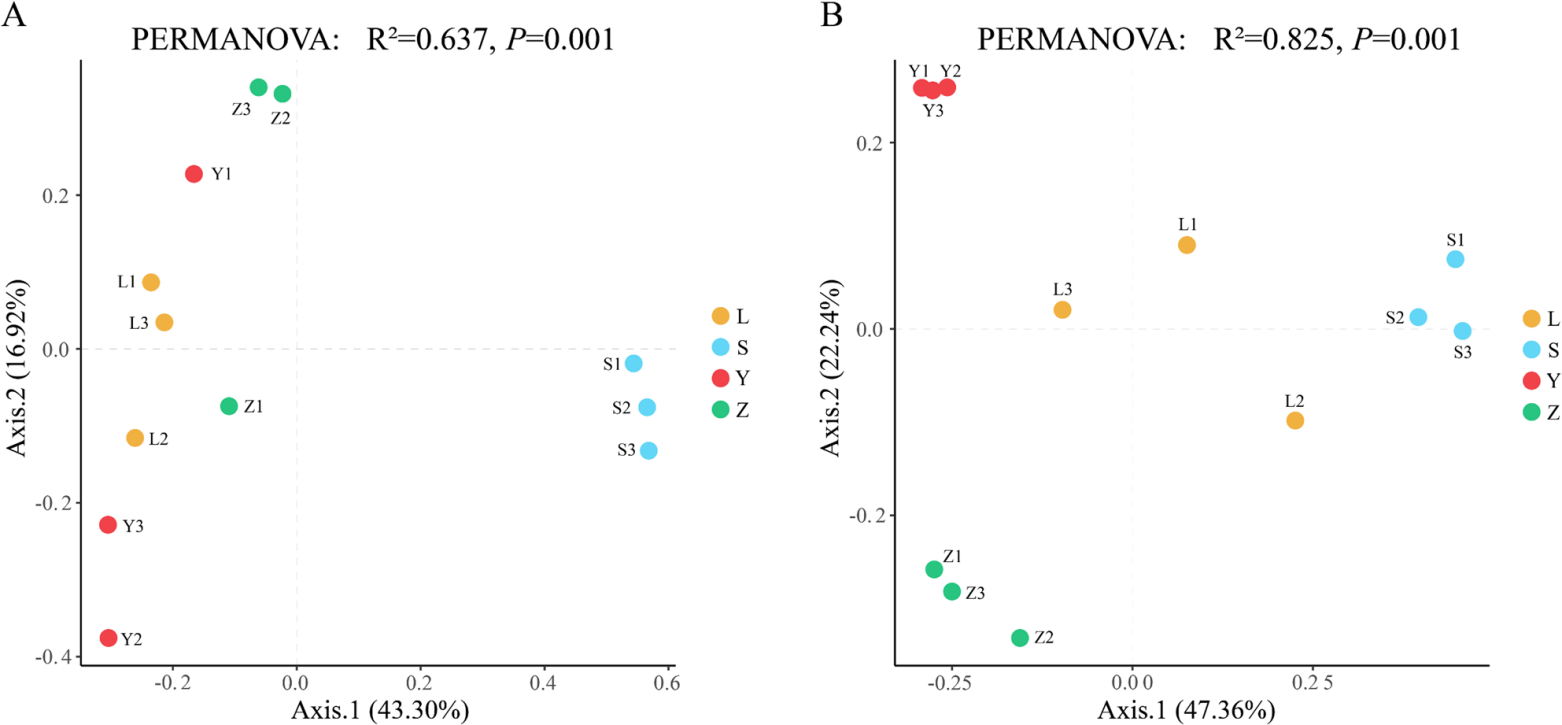
Principal coordinates analysis (PCoA) of microbial community beta diversity in Daqu samples. **A** Bacterial communities; **B** Fungal communities. L represents Luzhou, S represents Suining, Y represents Yibin, and Z represents Zigong.

Beta-diversity differences among the four Daqu samples were assessed using principal coordinates analysis (PCoA) based on Bray-Curtis distances (Fig. 5). For the bacterial community (Fig. 5A), the first two axes accounted for 43.30% (PC1) and 16.92% (PC2) of the total variance. Samples L and Y clustered closely, while S and Z were distinctly separated, indicating that the bacterial community structures of L and Y were similar but markedly different from those of S and Z. For the fungal community (Fig. 5B), PC1 and PC2 explained 47.36% and 22.24% of the variance, respectively. Sample Z was clearly distinct from the other three groups, highlighting a significant difference in fungal composition between Z and the remaining samples. Furthermore, β-diversity analysis based on PERMANOVA revealed significant differences in microbial community structure among regions, with bacteria showing R² = 0.637 and *P* = 0.001 (Fig. 5A), and fungi showing R² = 0.825 and *P* = 0.001. These results indicate that both bacterial and fungal communities exhibited strong regional differentiation in Daqu samples.

### Phylum-level composition of microbial communities in Daqu

**Fig. 6:**
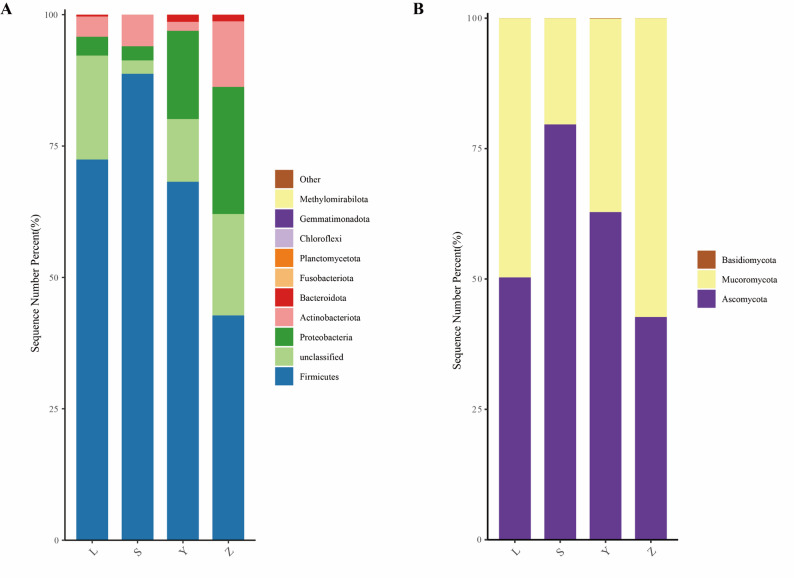
Phylum-level composition of microbial communities in Daqu samples. **A** Bacterial phyla; **B** Fungal phyla. Phyla with a relative abundance of less than 0.01% are categorized as “Others.” The letters L, S, Y, and Z represent Luzhou, Suining, Yibin, and Zigong, respectively.

Bacterial communities across the four Daqu samples showed broadly similar phylum-level profiles (Fig. 6A). Firmicutes dominated all samples, accounting for 42.77–88.75% of sequences, with the highest proportion in sample S (88.75%). Proteobacteria (2.70–24.18%) and Actinobacteria (1.70–12.48%) were also present, with sample Z exhibiting the highest abundance of both phyla. Bacteroidota (0.003–1.38%) was most abundant in sample Y, while unclassified reads were highest in samples L (19.77%) and Z (19.30%). Fungal communities displayed similar phylum-level composition across regions (Fig. 6B). Ascomycota was dominant (42.70–79.63%), peaking in sample S (79.63%), followed by Mucoromycota (20.35–51.29%), which was highest in sample Z (51.29%). The Basidiomycota phylum represented the smallest proportion, accounting for only 0.01–0.10%, with the highest value found in sample Y (0.10%).

### Genus-level composition of microbial communities in Daqu

**Fig. 7:**
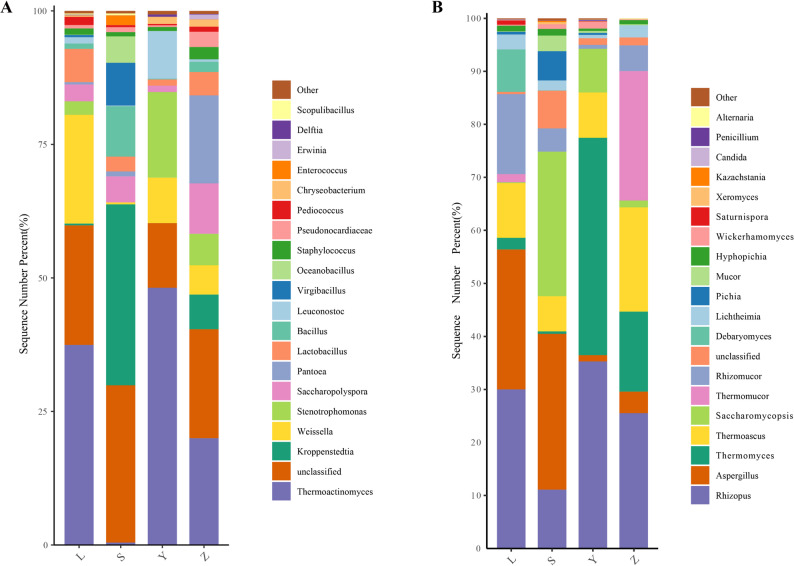
Genus-level composition of microbial communities in Daqu samples. **A** Bacterial genera; **B** Fungal genera. Genera with relative abundance < 0.01 % are grouped as "Others". The letters L, S, Y, and Z represent Luzhou, Suining, Yibin, and Zigong, respectively.

At the genus level, the top bacterial genera across all samples included *Thermoactinomyces* (0.45–48.16%), *Unclassified* (12.09–29.45%), *Kroppenstedtia* (0.01–33.88%), *Weissella* (0.37–20.37%), *Stenotrophomonas* (0.01–16.03%), *Saccharopolyspora* (1.19–9.45%), *Pantoea* (0.003–16.46%), *Lactobacillus* (1.17–6.23%), and *Bacillus* (0.13–9.44%) (Fig. 7A). Sample L was dominated by *Weissella* (20.37%) and Lactobacillus (6.23%), S by *Unclassified* (29.45%), *Kroppenstedtia* (33.88%), and *Bacillus* (9.44%), Y by *Thermoactinomyces* (48.16%) and *Stenotrophomonas* (16.03%), and Z by *Saccharopolyspora* (9.45%) and *Pantoea* (16.46%). For fungi, the ten most abundant genera were *Rhizopus* (11.10–35.28%), Aspergillus (1.19–29.40%), *Thermomyces* (0.44–41.00%), *Thermoascus* (6.64–19.68%), *Saccharomycopsis* (0.15–27.27%), *Thermomucor* (0–24.41%), *Rhizomucor* (0.74–15.11%), *Unclassified* (0.42–7.12%), and *Debaryomyces* (0–8.02%) (Fig. 7B). Sample L was enriched in *Rhizomucor* (15.11%), and *Debaryomyces* (8.02%), S in *Aspergillus* (29.40%) and *Saccharomycopsis* (27.27%), Y in *Rhizopus* (35.28%) and *Thermomyces* (41.00%), while Z had high *Thermoascus* (19.68%), and *Thermomucor* (24.41%). Notably, *Thermomucor* was only detected in L and Z.

### Differential taxa identified by LEfSe

**Fig. 8:**
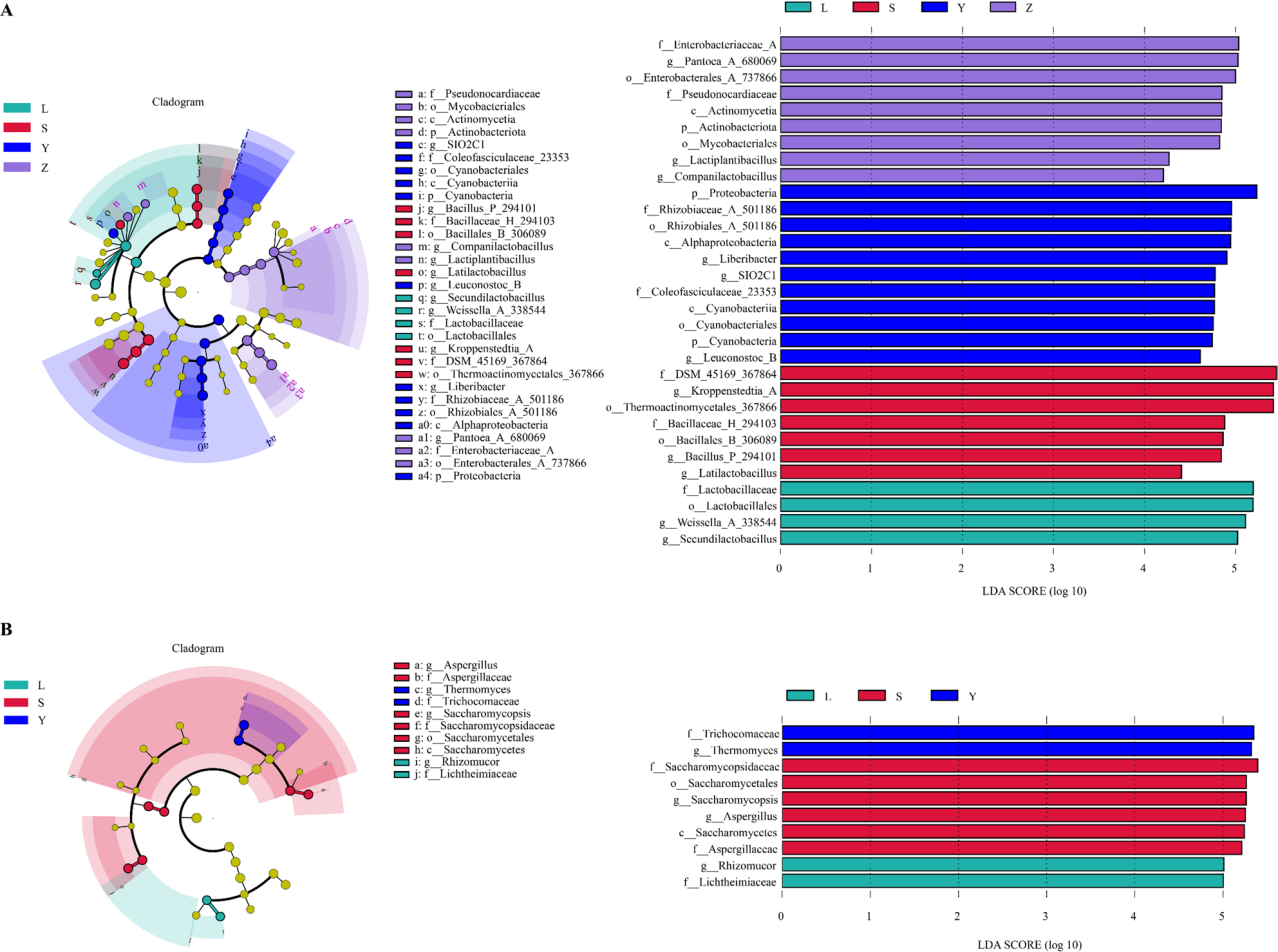
LEfSe biomarkers of microbial communities in Daqu samples. **A** Bacteria; **B** Fungi. Only taxa with an LDA score ≥ 4.0 are displayed. The letters L, S, Y, and Z represent Luzhou, Suining, Yibin, and Zigong, respectively.

LEfSe was used to identify taxa with significant regional differences, with an LDA threshold set at 4.0. The results are summarized in Fig. 8. For the bacterial community (Fig. 8A), 31 taxa showed significant regional discrimination. Sample Y exhibited the largest number of biomarkers (11 taxa). Region-specific indicator taxa included the family Lactobacillaceae for L, the family DSM_45169_367864 for S, the phylum Proteobacteria for Y, and the family Enterobacteriaceae_A for Z. For the fungal communities (Fig. 8B), ten taxa were identified as key biomarkers. Sample L was predominantly characterized by the genus *Rhizomucor*. Sample S contained the highest number of taxa (six), with the family Saccharomycopsidaceae being the most abundant. The primary indicator taxa in sample Y were from the family Trichocomaceae.

### Functional prediction of Daqu microbiota

**Fig. 9:**
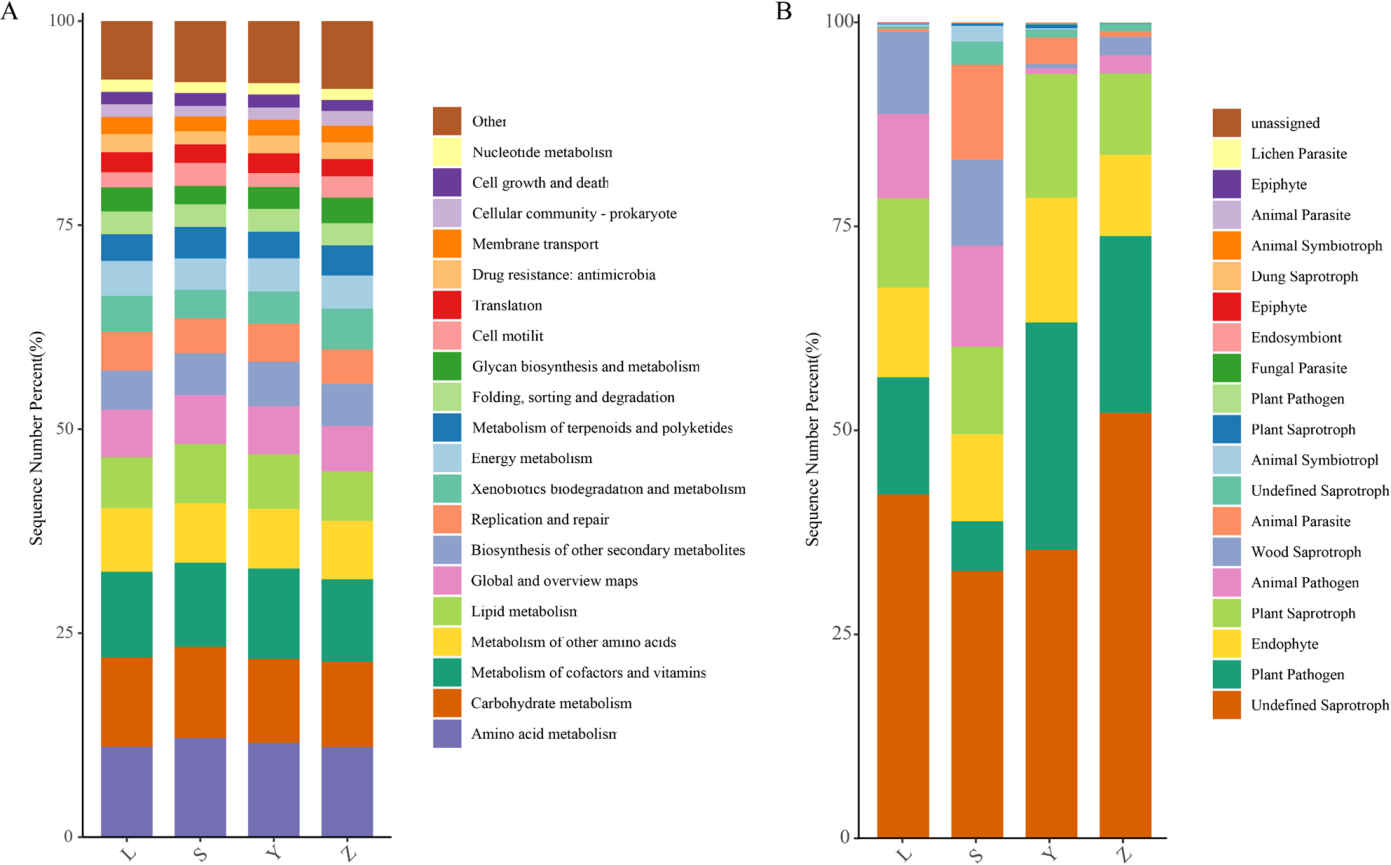
Predicted functional profiles of microbial communities in Daqu samples. **A** KEGG level-2 functional categories inferred from bacterial 16S rRNA data using PICRUSt2. **B** Fungal trophic modes classified according to FUNGuild. L, S, Y, and Z represent samples from Luzhou, Suining, Yibin, and Zigong, respectively.

Bacterial functional potential was inferred using PICRUSt2 against the KEGG database (Fig. 9A). Six level-1 categories were identified across all samples: Metabolism, Genetic Information Processing, Environmental Information Processing, Cellular Processes, Human Diseases, and Organismal Systems. At level-2, 46 pathways were detected, with amino acid metabolism, carbohydrate metabolism, and metabolism of cofactors and vitamins being the most abundant, indicating strong bacterial capacity for protein and carbohydrate processing. Notably, sample Z exhibited the lowest relative abundance in these pathways, suggesting weaker primary metabolic activity. Fungal guilds were predicted using FUNGuild (Fig. 9B), revealing four trophic modes: Pathotroph, Saprotroph, Symbiotroph, and Unassigned, with Saprotrophs predominating, particularly in sample L. Among the top 20 guilds, undefined saprotrophs, plant pathogens, endophytes, and plant saprotrophs were most frequent. Sample Z had a higher proportion of undefined guilds, whereas sample Y showed elevated abundances of plant pathogens and endophytes, reflecting potential regional functional differences.

### Correlation between dominant genera and physicochemical properties

**Fig. 10:**
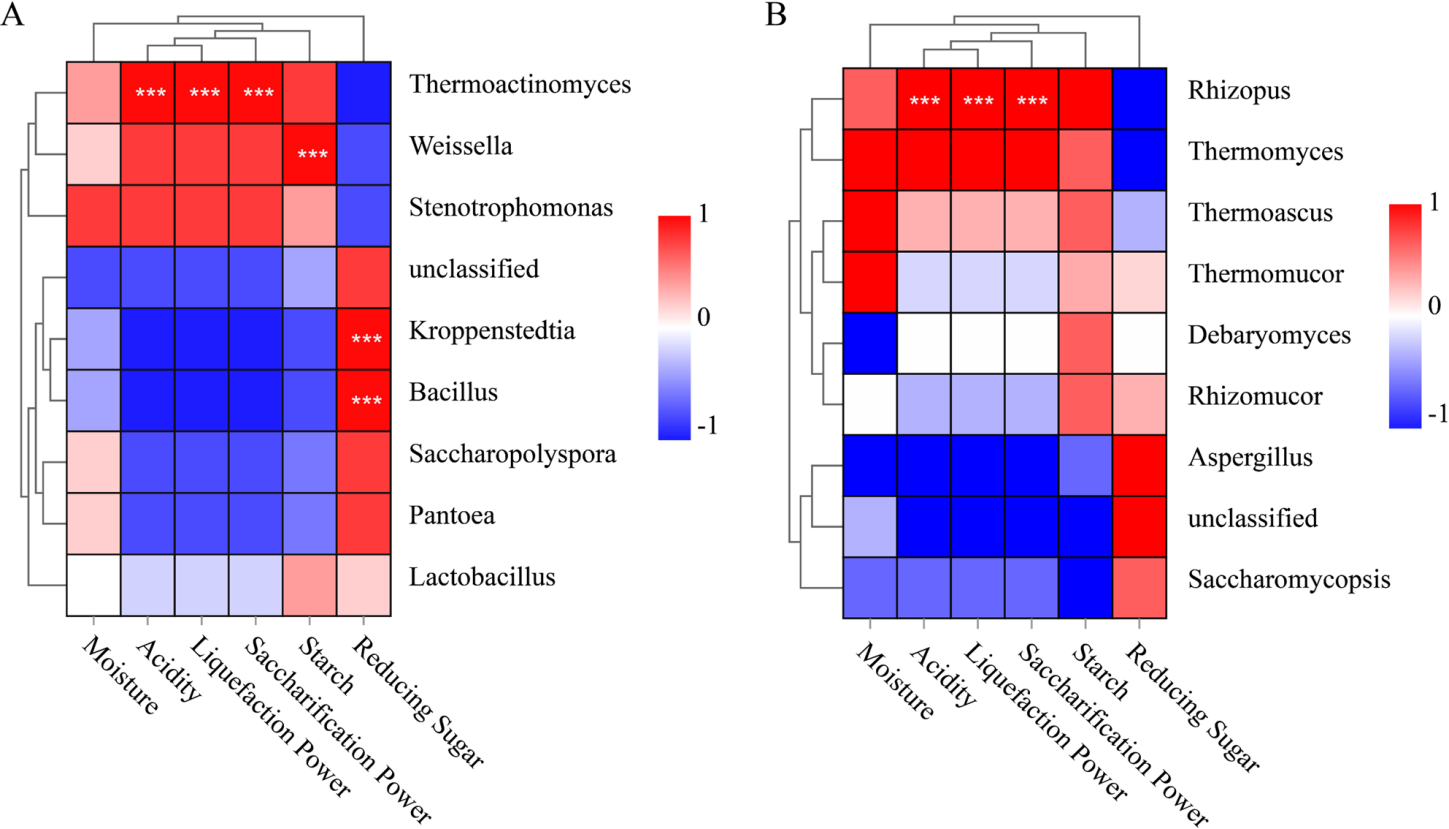
Spearman correlations between dominant microbial genera (relative abundance > 0.01%) and physicochemical properties of Daqu samples. **A** Bacteria; **B** Fungi. Positive correlations are indicated in red, and negative correlations are indicated in blue. Statistical significance is denoted as follows: *P* < 0.05 (*), *P* < 0.01 (**), *P* < 0.001 (***).

The correlation analysis between dominant genera and the physicochemical properties of Daqu samples is presented in Fig. 10. For bacterial genera, *Thermoactinomyces* exhibited a negative correlation with reducing sugar, while showing significant positive correlations with acidity, liquefaction power, and esterification power. *Weissella* was negatively correlated with reducing sugar but showed a significant positive correlation with starch content. *Kroppenstedtia* and *Bacillus* both showed significant positive correlations with reducing sugar, but were negatively correlated with acidity, liquefaction power, esterification power, and starch content. For fungal genera, *Rhizopus* demonstrated a negative correlation with reducing sugar and a highly significant positive correlation with acidity, liquefaction power, and esterification power. Unclassified fungal taxa showed a significant positive correlation with reducing sugar and a negative correlation with starch content.

## Discussion

### Regional variability in physicochemical properties of Daqu

Significant regional variations in the physicochemical properties of Daqu underscore the influence of local raw materials, fermentation conditions, and production techniques on Daqu quality (Li et al. 2023; Liu et al. 2022; Wang et al. 2021). The key physicochemical properties—moisture content, acidity, reducing sugar, starch content, liquefaction power, and esterification power—displayed notable differences among the four Daqu samples (L, S, Y, and Z). These findings are consistent with previous studies, which emphasize that the physicochemical properties of Daqu are critical in determining both fermentation efficiency and the sensory characteristics of the final Baijiu product (Deng et al. 2020; Mu et al. 2023).

Moisture content was significantly higher in Y and Z compared to S, suggesting that elevated moisture levels may promote the growth of moisture-dependent microorganisms, particularly under high-temperature fermentation conditions. This trend aligns with previous studies linking higher moisture content to enhanced microbial activity and improved fermentation performance (Wang et al. 2018). However, the absence of a significant difference between Y and Z implies that similar moisture levels may result in comparable microbial community structures, even when raw materials vary regionally (Yu et al. 2023). In particular, *Saccharomyces cerevisiae* and *Lactobacillus spp.* are likely to thrive under such conditions, contributing to fermentation efficiency and flavor development, though the specific microbial shifts could vary slightly with regional differences in other environmental factors.

Acidity, a key parameter in fermentation, varied significantly among the samples. Y exhibited the highest acidity, likely due to the abundant lactic acid production by dominant lactic acid bacteria such as *Lactobacillus* and *Weissella*, which thrive in the high-moisture, high-temperature environment of Y Daqu. This acidity plays a crucial role in ester formation, which is essential for Baijiu’s aromatic complexity (Deng et al. 2023; Zhang et al. 2024a, b, c). A clearer connection between acidity and downstream esterification efficiency has now been emphasized, as elevated acidity promotes ester production by enhancing microbial metabolism, leading to a more aromatic Baijiu. On the other hand, the lower acidity observed in L reflects a less acidic fermentation environment, producing a balanced yet distinct flavor profile characteristic of L’s regional Baijiu style (Hu et al. 2021a, b; Wang et al. 2022).

Reducing sugar content, a key determinant of fermentation efficiency, was highest in S sample, likely due to its lower moisture content, which may create a more concentrated sugar environment, thereby enhancing microbial fermentation activity. In contrast, the starch content in Y and L was significantly higher than in S, likely attributable to the raw materials used in these regions. Y and L, which rely more on wheat-based or mixed substrates, are known for their higher starch content (Jin et al. 2019; Zhang et al. 2024a, b, c). The absence of significant differences in starch content between Y and Z suggests that grain composition and fermentation temperature may play a more critical role than starch content alone in determining saccharification power.

Liquefaction power, an indicator of the ability to convert starch into fermentable sugars, was highest in Y, reflecting an optimal combination of high starch content and fermentation temperature, which likely supports the growth of amylolytic microbes. This is further supported by Y’s high esterification power, consistent with the high quality of Baijiu produced in this region. Conversely, the low esterification power observed in S may signal a less efficient fermentation process, potentially due to suboptimal microbial composition or fermentation conditions (Zhu et al. 2022).

Overall, the significant variation in these physicochemical characteristics across Daqu samples highlights the importance of regional factors such as raw material composition, fermentation temperature, and microbial community dynamics in determining Daqu quality. These results offer valuable insights into how optimizing Daqu’s physicochemical properties through controlled production conditions can influence the flavor and quality of the final Baijiu product.

### Alpha and beta diversity of microbial communities in Daqu

The alpha-diversity indices (Chao1, Shannon, and Simpson) revealed no significant differences across the four Daqu samples (Table 1), indicating that the overall richness and diversity of microbial communities were similar across regions. This finding aligns with previous studies suggesting that microbial diversity in solid-state fermentation is influenced by both intrinsic factors (e.g., raw materials) and external conditions (e.g., fermentation temperature), rather than a single dominant factor (Chen et al. 2025; Fu et al. 2021). PCoA based on Bray–Curtis distances further confirmed regional differences in microbial community composition (Fig. 5). For the bacterial community, samples from L and Y clustered together, while samples from S and Z were distinctly separated. This separation highlights a divergence in community structure, suggesting that the bacterial communities in L and Y share similar functional profiles, likely due to comparable environmental and substrate conditions (Mu et al. 2023). In contrast, S and Z exhibited distinct microbial signatures, which may be attributed to variations in raw material composition and fermentation conditions, including temperature and humidity (Zhang et al. 2024a, b, c). A similar separation was observed in the fungal community (Fig. 5B), with sample Z distinctly separated from the other three samples, indicating that the fungal communities in Z are markedly different from those in L, S, and Y. These results suggest that both bacterial and fungal communities in Daqu are influenced by regional factors, supporting the hypothesis that microbial composition varies significantly across different production regions (Zhu et al. 2025; Zong et al. 2024). While alpha diversity remained similar across all samples, the observed differences in beta diversity highlight the regional specificity of microbial communities in Daqu. These findings emphasize that although microbial richness and diversity do not always correlate with functional differences, the composition of bacterial and fungal communities significantly impacts Daqu’s fermentation properties, which in turn can affect the final flavor and quality of the Baijiu produced.

### Differences in microbial composition of Daqu at phylum and genus levels

The taxonomic composition of Daqu samples at both the phylum and genus levels revealed distinct regional patterns, reflecting the influence of local raw materials, fermentation conditions, and environmental microbiota on microbial community assembly (Deng et al. 2023; He et al. 2019).

At the phylum level, Firmicutes consistently dominated the bacterial communities, which aligns with their well-established roles in saccharification, acid production, and thermostability in traditional solid-state fermentation systems (Wang et al. 2022; Zhang et al. 2025). Sample S had the highest proportion of Firmicutes (88.75%), likely correlating with enhanced enzymatic activities, such as liquefaction and fermentation force, observed in this region’s Daqu. In contrast, samples Z and Y exhibited higher levels of Proteobacteria, Actinobacteria, and Bacteroidota, phyla associated with volatile compound transformation, nitrogen cycling, and secondary metabolite production. These phyla’s presence suggests their potential role in contributing to the flavor complexity of these Daqu types (Chen et al. 2025; Ma et al. 2025). The substantial fraction of unclassified taxa in samples L and Z indicates the presence of uncultured or poorly characterized microbes, which may represent novel functional resources for Baijiu fermentation and could be explored as part of the “microbial dark matter”. At the fungal phylum level, Ascomycota and Mucoromycota were dominant, consistent with their well-established amylolytic and proteolytic capacities (Wang et al., 2021). Sample S had the highest relative abundance of Ascomycota (79.63%), which likely contributes to the high saccharification efficiency observed in this region. In contrast, sample Z harbored a higher proportion of Mucoromycota (57.29%), highlighting the influence of raw material microbiota in shaping fungal community structure.

At the genus level, bacterial communities were dominated by a mix of thermotolerant (e.g., *Thermoactinomyces*, *Kroppenstedtia*), acid-producing (e.g., *Weissella*, *Lactobacillus*), and flavor-associated genera (e.g., *Stenotrophomonas*, *Pantoea*). The high abundance of *Weissella* and *Lactobacillus* in sample L aligns with its observed higher acidity, which may contribute to the accumulation of ester precursors. The presence of *Thermoactinomyces* and *Stenotrophomonas* in sample Y suggests their role in transforming amino acids and alcohols into flavor-active compounds (Ma et al. 2022a, b; Zhang et al. 2024a, b, c). Notably, sample Z was enriched in *Saccharopolyspora* and *Pantoea*, genera known for secondary metabolite production, which could impart unique aromatic traits to Daqu from this region. Fungal genera also varied significantly across samples. *Rhizopus*, *Aspergillus*, and *Thermomyces* were core genera with known contributions to starch degradation and flavor development (Li et al. 2016; Mu et al. 2023). The co-dominance of *Rhizopus* and *Thermomyces* in sample Y likely explains its high saccharification and fermentation capacity. *Saccharomycopsis*, abundant in sample S, is implicated in ethyl acetate synthesis and may contribute to regional aroma characteristics. The exclusive detection of *Thermomucor* in L and Z underscores the influence of temperature and substrate conditions on fungal succession.

These findings emphasize that while core phyla and genera dominate across all samples, the regional distribution of microbial taxa plays a crucial role in shaping the physicochemical properties and flavor of strong-aroma Daqu. The presence of unclassified taxa offers an exciting opportunity to explore “microbial dark matter” and its potential in Baijiu fermentation, contributing to the distinct terroir of Baijiu production in southwestern China.

### Biomarker taxa and functional specialisation

LEfSe analysis identified distinct sets of bacterial and fungal biomarkers for each Daqu region. The enrichment of *Lactobacillaceae* in L aligns with previous reports suggesting that high-lactic starter stacks favor *Lactobacillus*, which enhances acidity regulation and ester precursor formation in strong-aroma Baijiu (Li et al. 2024; Zhu et al. 2022). In contrast, S was characterized by the family *DSM_45169_367864*, a thermophilic actinomycete lineage previously isolated from medium-temperature wheat–pea starters. This family has been linked to robust amylase production, which supports efficient saccharification (Li et al. 2017; Zhang et al. 2024a, b, c). Y displayed an over-representation of Proteobacteria, including genera such as *Pantoea* and *Stenotrophomonas*, which are known to contribute alcohol dehydrogenases and esterases, enhancing fruity notes in Chinese Baijiu (Zhang et al. 2024a, b, c; Zong et al. 2024). The dominance of Enterobacteriaceae_A in Z mirrors findings from multi-grain, cellar-aged starters, where oxygen-limited niches promote facultative anaerobes capable of higher-alcohol synthesis (Wang et al. 2018).

Fungal biomarkers further emphasize regional specialization. Sample L was mainly dominated by *Rhizomucor*, a genus important in the fermentation of Daqu used for Baijiu production. This fungus likely plays a role in breaking down organic matter during fermentation (Deng et al. 2025; Liu et al. 2024). Y was marked by Trichocomaceae (e.g., *Aspergillus*, *Thermomyces*), taxa renowned for esterase and protease secretion, which aligns with Y’s high esterification activity (Hu et al. 2017). Sample S exhibited the highest diversity, with Saccharomycopsidaceae being the most abundant family. This indicates a more complex microbial community, which may enhance Baijiu fermentation and contribute to flavor development (Ma et al. 2022a, b). In sample Y, Trichocomaceae was the dominant family. Known for its role in decomposing organic materials, this group may play a key part in the fermentation process and the final flavor profile of Baijiu.

Collectively, these biomarker taxa reinforce the observed physicochemical patterns (e.g., high acidity in L, strong saccharification in Y) and serve as microbial indicators for authenticating regional Daqu. These findings also offer targets for selective enrichment or attenuation in future precision-engineered starters, aimed at harmonizing traditional flavors with enhanced process consistency.

### Predicted functional potential mirrors biochemical assays

PICRUSt2 projection mapped 16 S rRNA data to six primary KEGG categories, with amino-acid metabolism, carbohydrate metabolism, and metabolism of cofactors and vitamins showing the highest secondary-level abundances across all samples. These profiles align with the requirements of solid-state Baijiu fermentation, where proteolysis and starch hydrolysis provide fermentable substrates and aroma precursors (Li et al. 2025; Ma et al. 2022a, b). Notably, sample Z exhibited the lowest relative abundance in these pathways, correlating with its reduced acidity. This suggests that the cool, cellar-aged fermentation process favors the selection of bacteria with lower amylolytic and proteolytic activity, which in turn may contribute to the lower acid production observed in this sample. This trend has been previously observed in multi-grain starters fermented under similar conditions. (He et al. 2024; Jin et al. 2019). Fungal annotation *via* FUNGuild identified saprotrophs as the dominant guild, particularly in L, where their high prevalence supports efficient macromolecule degradation. Saprotrophic genera such as Rhizopus and Aspergillus are well-known for secreting amylases, proteases, and esterases critical for flavor formation (Li et al. 2016; Wang et al. 2021). In contrast, Y exhibited the highest proportions of plant pathogens and endophytes, likely introduced with the wheat raw material during starter molding, a trend similarly reported in other wheat-based fermentations (Liu et al. 2022; Yu et al. 2023). The high proportion of undefined guilds in Sample Z indicates the presence of uncharacterized fungal taxa, which may shape its distinctive flavor profile or pose challenges to quality control. This underscores the necessity of further isolation and metagenomic analyses to elucidate their functional roles. Subsequent metagenomic studies could provide deeper insights into these undefined taxa and their functional contributions to Daqu fermentation.

### Microbe–property correlations clarify mechanistic links

Spearman correlation analysis revealed mechanistic links between microbial communities and the physicochemical properties of Daqu (He et al. 2023). The negative correlation between *Thermoactinomyces* and reducing sugar suggests its involvement in the utilization of sugar derivatives or complex carbohydrate breakdown during fermentation, consistent with its known role in protein and carbohydrate metabolism in fermented foods (Chen et al. 2022; Wu et al. 2020). Positive correlations of *Thermoactinomyces* with acidity, liquefaction power, and esterification power suggest its contribution to organic acid production and enzymatic activities, enhancing fermentation, as observed in other fermentation systems (Zhang et al. 2025). *Bacillus* and *Kroppenstedtia*, positively correlated with reducing sugar, are likely involved in the early stages of fermentation when sugar is abundant (Ma et al. 2022a, b; Wang et al. 2018). However, their negative correlations with esterification power and starch content suggest a more complex role in limiting ester formation and starch hydrolysis, potentially affecting the texture and flavor profile of Daqu (Liu et al. 2024; Zong et al. 2024).

*Rhizopus*, which showed a strong positive correlation with acidity, liquefaction power, and esterification power and a negative correlation with reducing sugar, likely contributes to ester formation, enhancing the characteristic flavors of Daqu (Fu et al. 2021; He et al. 2023). The unclassified fungal genera also played a significant role in sugar metabolism and starch breakdown, indicating that less-studied microbial taxa could be contributing to Daqu’s unique characteristics. The fungal genus *Saccharomycopsis* was negatively correlated with starch content, indicating its role in starch degradation, essential for improving fermentation efficiency (Song et al. 2019; Wang et al. 2021). These findings are consistent with research on microbial interactions in fermented foods, where unclassified taxa often play vital roles in fermentation dynamics (Hu et al. 2021a, b; Wang et al. 2021).

Overall, the correlation between microbial communities and Daqu’s physicochemical properties highlights the critical role of microbial diversity in shaping the fermentation process and influencing product quality. Future research should focus on functional genomics to explore the metabolic pathways of these key microbial genera and their impact on fermentation outcomes.

## Conclusion

This study identified significant regional variations in the physicochemical properties and microbial communities of strong-aroma Daqu from southwestern China. Specifically, Y exhibited high saccharification potential, while Z showed higher acidity, highlighting the influence of regional factors on fermentation outcomes. While Firmicutes and Ascomycota predominated across all samples, the genus-level composition and functional profiles revealed significant regional distinctions. The LEfSe biomarkers and functional predictions highlighted the specific fermentation conditions and raw material usage in each region, providing a deeper understanding of the microbial functions driving these variations. Correlation analysis further confirmed connections between key microbial taxa and essential quality indicators, such as acidity, saccharification, and liquefaction power. These findings not only provide valuable insights into the regional characteristics of Daqu but also offer potential microbial targets for optimizing fermentation quality, developing standardized Daqu starter cultures, and preserving region-specific flavor signatures. Furthermore, the study highlights the potential for industrial microbial resource development to enhance Baijiu production. Future research should focus on validating the predicted functions through metagenomics and metabolomics, conducting controlled fermentation trials with identified biomarker taxa, and exploring the role of unclassified microbial taxa in shaping Daqu’s unique characteristics.

## Supplementary Information

Below is the link to the electronic supplementary material.

Supplementary Material 1

## Data Availability

The data that support the findings of this study are available from the corresponding author upon reasonable request.
