# Supplementary material for: Comparative analysis of microbial communities and physicochemical attributes of strong-aroma Daqu from Southwestern China
Source: Bioresour Bioprocess. 2026 Jan 27;13(1):8. doi: 10.1186/s40643-025-00997-z (PMC12835487; doi:10.1186/s40643-025-00997-z)
Supplement: Supplementary file 1 — Supplementary Material 1 [file 40643_2025_997_MOESM1_ESM.docx]

**Table S1. Regional Daqu characteristics**

| Region | Primary substrates | Production temperature regime | Storage/processing notes |
| --- | --- | --- | --- |
| L | Sorghum-based | High-temperature | Dry, ventilated; avoid sun |
| S | Wheat-pea blend | Medium-temperature | Ventilated racks; routine checks for uniformity |
| Y | Pure wheat | Gradient temperature control | Cool, ventilated room; slow, even drying |
| Z | Multi-grain blend | Medium-temperature (typical) | Stored in earthen cellars |

Note: L represents Luzhou, S represents Suining, Y represents Yibin, and Z represents Zigong

**Figure S1 Rarefaction curves of Daqu samples**


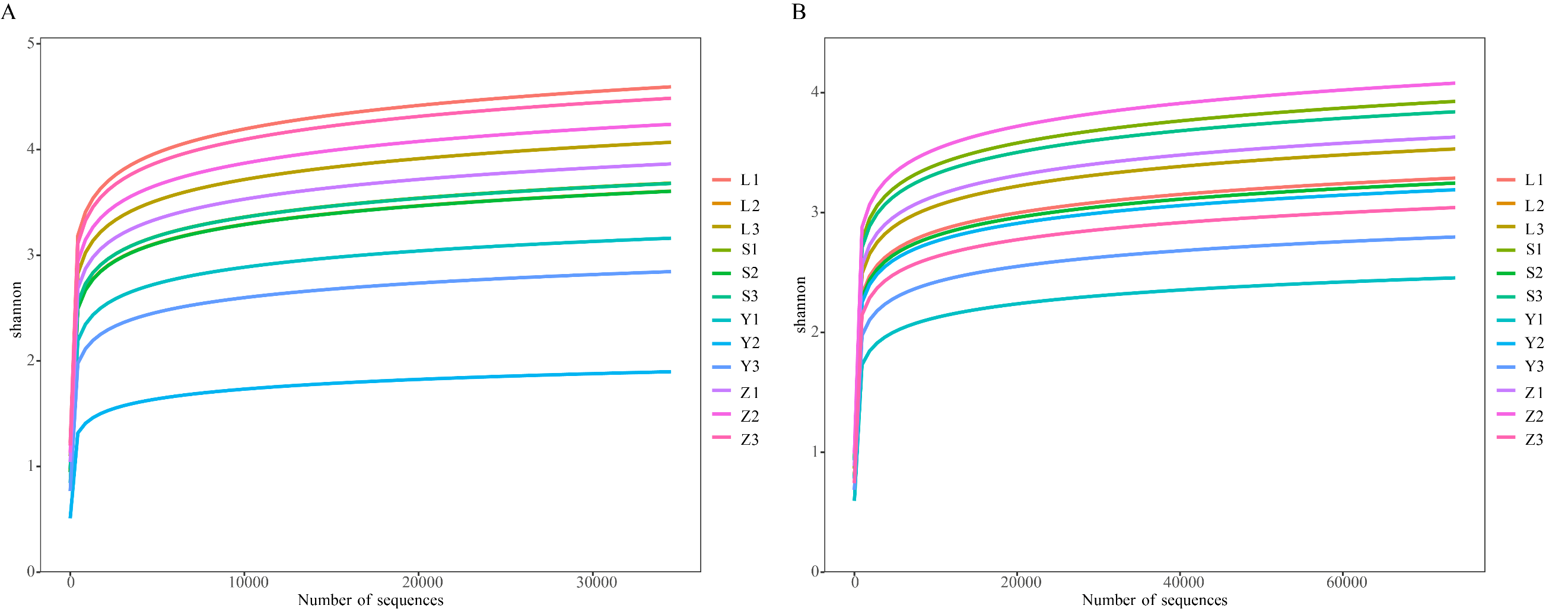


Note: L represents Luzhou, S represents Suining, Y represents Yibin, and Z represents Zigong. (A) Bacteria; (B) Fungi.
